# Intravenous diclofenac and orphenadrine for the treatment of postoperative pain after remifentanil-based anesthesia: A double-blinded, randomized, placebo-controlled study.

**DOI:** 10.1007/s00508-022-02131-x

**Published:** 2022-12-28

**Authors:** Sebastian Zeiner, Thomas Haider, Oliver Zotti, Katrin Thüringer, Petra Höbart, Oliver Kimberger, Erich Knolle

**Affiliations:** 1 Department of Anesthesia, General Intensive Care, and Pain Management, Medical University of Vienna, Währinger Gürtel 18–20, Vienna, Austria; 2 Department of Orthopedics and Trauma-Surgery, Medical University of Vienna, Vienna, Austria; 3 Ludwig Boltzmann Institute for Digital Health and Patient Safety (LBI-DHPS), Vienna, Austria; 4 Outcomes Research Consortium, Cleveland, OH USA

**Keywords:** Pain management, Postoperative care, Postoperative pain control, Multimodal pain, Non-steroidal anti-inflammatory drugs (NSAIDs)

## Abstract

**Background:**

Postoperative intravenous diclofenac reduces patient opioid demand and is commonly used in surgical units. Orphenadrine is mainly used in combination with diclofenac for musculoskeletal injuries and postoperative pain control. The objective of this study was to compare the analgesic efficacy of diclofenac-orphenadrine, diclofenac alone and saline.

**Methods:**

We performed a double-blind, randomized, placebo-controlled, parallel-group, single-center clinical study investigating the opioid-sparing effect of a combination of diclofenac and orphenadrine versus diclofenac alone versus isotonic saline solution. Initially 72 patients were included and received total intravenous anesthesia during cruciate ligament surgery. All patients were postoperatively treated with a patient-controlled analgesia (PCA) device containing hydromorphone. Pharmacological safety was assessed by laboratory parameters, vital signs, and delirium detection scores.

**Results:**

There was no significant difference between the groups in cumulative dose of PCA analgesics required after 24 h postsurgery, with 5.90 mg (SD ± 2.90 mg) in the placebo group, 5.73 mg (SD ± 4.75 mg) in the diclofenac group, and 4.13 mg (SD ± 2.57 mg) in the diclofenac-orphenadrine group. Furthermore, there was no significant difference between the groups in cumulative dose of PCA analgesics required 2 h postsurgery (*n* = 65). Mean dose of hydromorphone required after 2 h was 1.54 mg (SD ± 0.57 mg) in the placebo group, 1.56 mg (SD ± 1.19 mg) in the diclofenac-only group, and 1.37 mg (SD ± 0.78 mg) in the diclofenac-orphenadrine group. However, when comparing the diclofenac-orphenadrine group and the diclofenac group combined to placebo there was a significant reduction in PCA usage in the first 24 h postsurgery. In total, there were 25 adverse events reported, none of which were rated as severe.

**Conclusion:**

Orphenadrine-diclofenac failed to significantly reduce postoperative opioid requirements. However, in an exploratory post hoc analysis the diclofenac-orphenadrine and the diclofenac group combined versus placebo showed a tendency to reduce opioid demand in postoperative pain control. Further research is required to determine the value of orphenadrine as an adjuvant in a multimodal approach for postoperative pain management.

## Introduction

The benefits of effective pain management after surgery and the favorable effect on fast recovery and outcome are well documented [1]. A multimodal approach including opioids and nonsteroidal anti-inflammatory drugs (NSAIDs) in particular coxibs or paracetamol (acetaminophen) is recommended [2, 3]. Intravenous diclofenac has an established role in the treatment of acute and chronic pain as well as in postoperative pain and has been in use for several decades in Europe [4–6]. Diclofenac is a NSAID which inhibits cyclooxygenase (COX) isoenzymes 1 and 2, thereby reducing the production of prostaglandins peripherally as well as in the central nervous system [7]. The side effect profile of diclofenac can be explained by the inhibition of COX 1 and 2 as well. The inhibition of the production of prostaglandins and thromboxane potentially leads to gastrointestinal, hematological, cardiovascular and renal adverse events [4, 8]. To date, the discussion concerning cardiovascular risk of short-term diclofenac use is still ongoing. Large randomized controlled trials would be required to settle this debate but are unlikely to be conducted due to the disproportionate expense-benefit ratio. Thus, based on observational data the use of diclofenac in cardiovascular risk patients is not recommended at the moment [9–12]. Furthermore, according to a meta-analysis there are several case reports on diclofenac-related adverse psychiatric events [13].

Orphenadrine is a centrally acting muscle relaxant initially used for the treatment of Parkinson’s disease. In addition, it has anticholinergic and antihistaminic properties and a potential analgesic efficacy via an antagonistic effect on N‑methyl-D-aspartate (NMDA) receptors, as well as inhibiting effects on the norepinephrine re-uptake system. The exact mechanisms of its analgesic effects are unclear but a block of sodium channels could be a possible mechanism in its analgesic efficacy [14–17]. Currently, orphenadrine is mainly used in combination with paracetamol or diclofenac for musculoskeletal injuries like strains, sprains and acute lower back or neck pain [18]. Several studies exist for the combination of diclofenac and orphenadrine in postoperative as well as emergency department settings which prove superiority over placebo but a potential superiority over diclofenac alone remains yet to be determined [19–21]. Furthermore, a hypothetical influence of orphenadrine on possible hyperalgesic effects of intraoperative remifentanil is unknown [22, 23]. Because of its mild anticholinergic effects, psychiatric side effects, such as hallucinations, agitation and mental confusion especially in old people have been described [24, 25].

The objective of this study was to investigate the potential opioid-sparing effect of a combination of diclofenac and orphenadrine versus diclofenac alone. We hypothesized that the addition of orphenadrine might also prevent opioid-induced secondary hyperalgesia after intraoperative use of potent opioids such as remifentanil.

## Methods

### Study design

The study was performed at the Vienna General Hospital, which is also the site of the Medical University of Vienna and provides more than 50,000 surgeries per year. The study was conducted as a double-blind, randomized, placebo-controlled, parallel-grouped, single-center clinical study.

### Ethics

This study was conducted in accordance with the declaration of Helsinki and the good clinical practice guidelines. The study protocol as well as the written consent form were approved (EK 1494/2016) by the Ethics Committee of the Medical University of Vienna (Chairperson: Univ.-Doz. Dr. Martin Brunner; Borschkegasse 8b/E06, 1090 Vienna, Austria) on 24 August 2016. Written informed consent was obtained from all study participants.

### Study population

Included were patients undergoing elective cruciate ligament surgery. Every patient received a standard of care total intravenous anesthesia consisting of an induction with propofol, remifentanil and, if relaxation required, rocuronium followed by a maintenance with remifentanil (0.15–0.25 μg⋅kg^−1^per minute) and propofol (4–7 mg⋅kg^−1^ per hour). Maintenance dose was adapted by the attending anesthesiologist according to heart rate, blood pressure and other clinical signs of stress or pain.

Sample size calculations: from chart reviews, we derived a range of 4–6 mg hydromorphone usage within 24 h for patients with no prophylactic diclofenac intraoperatively, diclofenac, and diclofenac plus orphenadrine administration intraoperatively with a standard deviation of 2 mg. With an a‑error of 0.05 and a power (1-b) of 0.85, and a 1:1:1 allocation ratio 66 patients overall were calculated and 10% were added to compensate for study dropouts for ANOVA analysis, leading to 72 patients.

### Study intervention

**Fig. 1:**
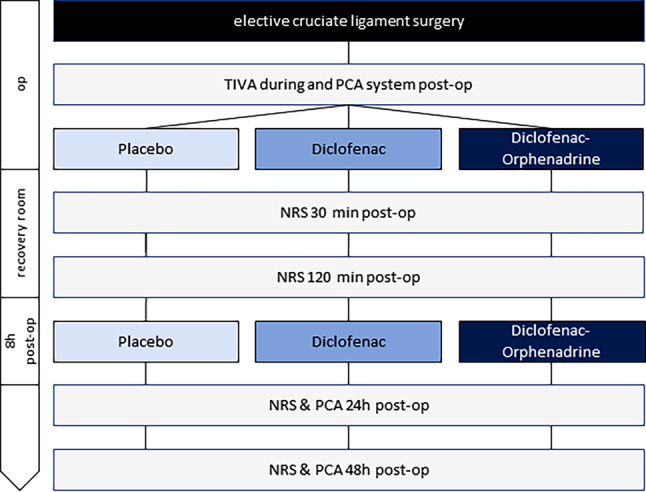
Flowchart of the study protocol. *TIVA* total intravenous anesthesia, *PCA* patient-controlled analgesia, *VAS* visual analogue scale

Patients were randomly allocated in a 1:1:1 ratio to 1 of 3 study arms and received either 2×250 ml solutions of 75 mg diclofenac combined with 30 mg of orphenadrine, 2×250 ml solutions of 75 mg diclofenac or 2×250 ml solutions of isotonic saline in a double-blind setting. Randomization of the study patients in a 1:1:1 randomization ratio took place presurgery, using the web-based randomizer provided by the Institute for Medical Informatics, Statistics and Documentation, Medical University of Graz (Graz, Austria, https://www.randomizer.at/). Study medication was prepared in advance by the hospital pharmacy and labelled only with a group number and date of preparation. Only the hospital pharmacy had knowledge about which group corresponded with which study arm. Consequently, participants, care providers as well as those assessing outcomes were blinded. The first infusion of study medication was given prior to emergence from anesthesia, immediately after completion of the ligament reconstruction and the second infusion 8 h ± 30 min later (Fig. 1). All patients were provided with a patient-controlled analgesia (PCA) device (Vygon PCA-System, Vygon, Aachen, Germany). The PCA system contained 20 mg hydromorphone in 50 ml isotonic saline for intravenous self-administration. The PCA system parameters were adjusted to a single bolus volume of 0.5 ml and a flow regulator limiting the bolus chamber refilling to 0.5 ml/5 min resulting in a maximum dose of 0.2 mg hydromorphone every 5 min. Included were patients undergoing elective arthroscopic cruciate ligament reconstruction aged 18–85 years. Exclusion criteria were known hypersensitivity to one of the investigated compounds, congestive heart failure classified as NYHA 2 or higher, ischemic heart disease, peripheral arterial occlusive disease, cerebrovascular diseases and significant risk factors for cardiovascular events, namely hypertension, hyperlipidemia, type 2 diabetes and smoking more than 10 cigarettes per day. Furthermore, pregnancy was ruled out via a pregnancy test (human chorionic gonadotropin (hCG) urin level) in female patients prior to enrolment in this study.

### Statistics

The primary outcome of the study was the total dose of PCA analgesics required 24 h after surgery. The secondary outcomes were PCA analgesics required 2 h after surgery, pain intensity measured by visual analogue scales (VAS) 2 h and 24 h after surgery.

Exploratory outcomes: furthermore, the local and systemic tolerability and safety of the clinical study medications were assessed, as were possible neurological side effects using the delirium detection score 2 h and 24 h after the first infusion [26]. Overall pharmacological safety was assessed via laboratory parameters, vital signs and questionnaires for the first 48 h postsurgery. Demographic information including gender, age, and body mass index (BMI) was collected for all patients.

Results are reported as means with standard deviation (SD). Dichotomous outcomes are reported as absolute (*n*) and relative values (%). Continuous outcomes are reported as mean with standard deviation or median with interquartile range (IQR). The one-way analysis of variance (ANOVA) was used to determine whether there were any statistically significant differences between the three study groups. The χ^2^-test, Fisher’s exact test and Student’s t‑test were utilized to analyze categorical and continuous variables between two groups and a *p*-value of less than 0.05 was considered statistically significant. The SPSS (IBM SPSS Statistics Version 24, IBM, Armonk, NY, USA) and Graph Pad Prism 6 (GraphPad Software, San Diego, CA, USA) were used for all analyses.

## Results

**Fig. 2:**
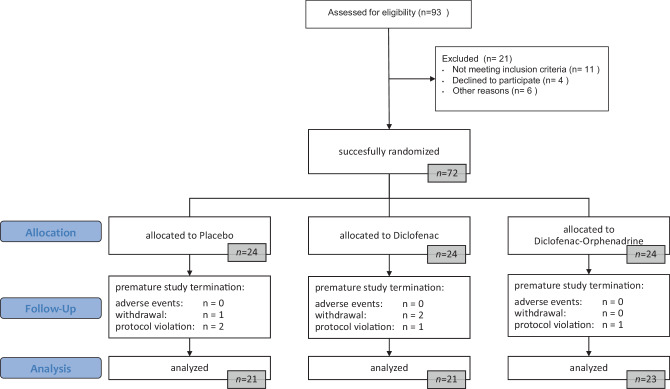
Patient flowchart (Consort flow diagram, adapted)

Between February 2018 and May 2019, 72 patients scheduled for cruciate ligament surgery were randomized to either the placebo, the diclofenac only or the diclofenac-orphenadrine arm. Treatment was initiated in all 72 patients. One patient in the placebo and one patient in the diclofenac-orphenadrine group dropped out due to intraoperative changes in surgical procedure. In both cases only an arthroscopic meniscus repair was performed. One patient of the diclofenac and one patient of the placebo group were excluded due to a damaged PCA system leading to a deviation from the study protocol and three patients, two in the diclofenac group and one in the placebo group, withdrew their consent after surgery. Therefore, 65 patients completed the study, 21 in the placebo group, 21 in the diclofenac only group and 23 in the diclofenac-orphenadrine group (Fig. 2).

Mean study population age was 31.4 years (SD = 9.25) with 27.7% female patients and the mean body mass index (BMI) was 24.96 = kg/m^2^ (SD = 3.56). Detailed demographics for each group are shown in Table 1. Mean doses of propofol were comparable between all groups with 5.81 mg⋅kg^−1^ per hour (SD = 0.83) in the placebo group, 6.12 mg⋅kg^−1^ per hour (SD = 1.07) in the diclofenac only group and 5.55 mg⋅kg^−1^ per hour (SD = 0.92) in the diclofenac-orphenadrine group. Moreover, remifentanil doses were similar with 0.281 μg⋅kg^−1^ per minute (SD = 0.056) in the placebo group, 0.297 μg⋅kg^−1^ per minute (SD = 0.068) in the diclofenac only group and 0.269 mcg.kg^−1^ per minute (SD = 0.083) in the diclofenac-orphenadrine group.

**Table 1:** Patient demographics and baseline characteristics

			Placebo	Diclofenac	Diclofenac-orphenadrine
Sex	f	*n*	5	3	10
		%	23.8	14.3	43.5
	m	*n*	16	18	13
		%	76.2	85.7	56.5
Age (years)	Mean		31	32	31
	SD		9	10	9
Weight (kg)	Mean		75.4	78.3	76.0
	SD		11.9	8.6	15.2
Height (cm)	Mean		174	179	172
	SD		9	7	9
BMI (kg/m^2^)	Mean		25.0	24.4	25.5
	SD		3.0	2.8	4.6
Smoking status	Never	*n*	16	11	16
	Past	*n*	0	0	1
	Current	*n*	5	10	6
Duration of surgery (min)	Mean	min	101	92	95
	SD		28	30	32

*SD* standard deviation, *f* female, *m* male, *BMI* body mass index

**Fig. 3:**
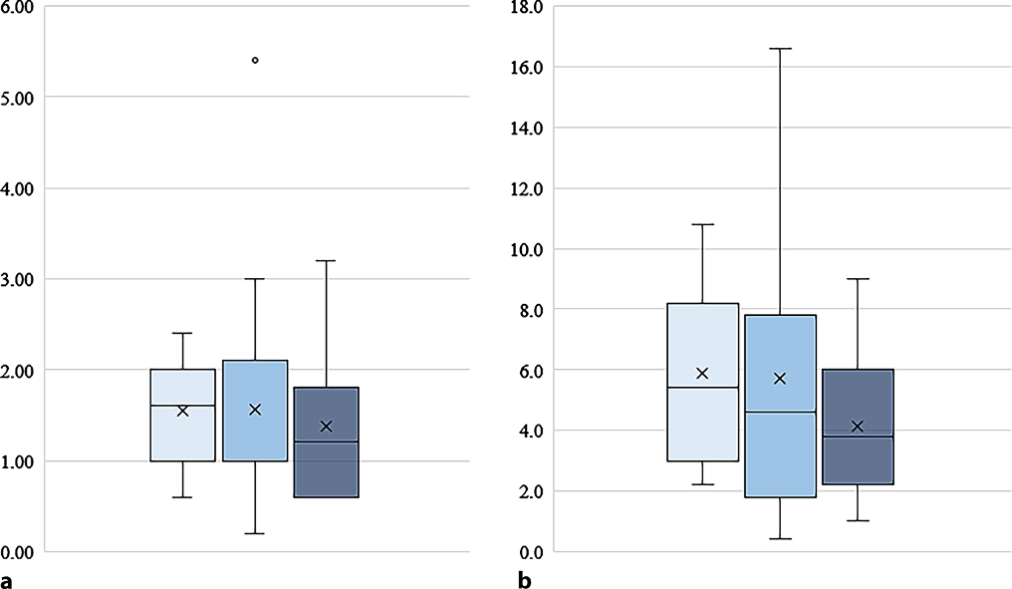
PCA hydromorphone requirement in mg 2 h (**a**) and 24 h (**b**) postoperatively for the placebo group (*light blue*), the diclofenac group (*blue*) and the diclofenac-orphenadrine group (*dark blue*). *PCA* patient controlled analgesia

There was no significant difference between the groups in total dose of PCA analgesics required over 24 h postsurgery. The means were 5.90 mg (SD = 2.90) in the placebo group, 5.73 mg (SD = 4.75) in the diclofenac group, and 4.13 mg (SD = 2.57) in the diclofenac-orphenadrine group. In post hoc analysis, when excluding outliers, namely 2 datapoints of the diclofenac group lying outside the Q3 + 1.5 IQR, there was a significant difference in PCA analgesics required over 24 h when comparing the diclofenac-orphenadrine and the diclofenac only groups combined (mean 4.34 mg (SD = 2.89)) to the placebo group (mean 5.89 mg (SD = 2.90); *p* = 0.049) (Fig. 3).

However, there was no significant difference between the groups in total dose of PCA analgesics required within 2 h postsurgery. Mean dose of hydromorphone required within 2 h was 1.54 mg (SD = 0.57) in the placebo group, 1.56 mg (SD = 1.19) in the diclofenac only group and 1.37 mg (SD = 0.78) in the diclofenac-orphenadrine group.

Pain intensities measured by VAS did not significantly differ between the three groups. Neither 30 min after the first infusion (50.33 mm (SD = 19.45) placebo, 49.52 mm (SD = 24.59) diclofenac only, 57.35 mm (SD = 12.97) diclofenac-orphenadrine, ns) nor after 120 min (30.52 mm (SD = 13.88) placebo, 29.05 mm (SD = 14.46) diclofenac, 29.70 mm (SD = 13.42) diclofenac orphenadrine) nor after 24 h (28.57 mm (SD = 21.92) placebo, 25.71 mm (SD = 13.54) diclofenac, 28.48 mm (SD = 16.48) diclofenac-orphenadrine).

In total, there were 25 adverse events reported, none of which were rated as severe. Nausea was comparable in all groups with 23.8% (*n* = 5) in the placebo group, 33.3% (*n* = 7) in the diclofenac only group and 26.1% (*n* = 6) patients in the diclofenac-orphenadrine group.

None of the patients tested positive for delirium using the delirium detection score, neither 2h after the operation nor the day after.

## Discussion

In the present study, there was no significant difference for the postoperative opioid PCA requirements between the diclofenac-orphenadrin, the diclofenac and the placebo group for the treatment of postoperative pain in patients receiving cruciate ligament surgery. However, a distinct trend towards a reduction of hydromorphone requirement in the first 24 h postsurgery was found when administering diclofenac-orphenadrine, albeit being non-significant. Furthermore, our findings are in line with existing literature regarding high interindividual differences of opioid effectiveness as well as responsiveness to pain and a wide variety in postoperative opioid demand after arthroscopic cruciate ligament surgery [27, 28].

Overall, subjective pain intensity was similar in all groups. Due to adequate dosing of rescue medication a difference in pain intensity between the groups was not to be expected. Following the development of PCA systems with the ability to record delivered dose in the early 1970s reduction of opioid consumption became a cornerstone for the assessment of pain medication [29, 30]. Our finding that pain intensity was at a satisfactory level throughout the observation period underlines the well-established benefits of PCAs [23].

To the best of our knowledge there previously were three studies examining the intravenous use of a diclofenac-orphenadrine combination in a postoperative setting. However, two of these studies were published in Hungarian and in Czech, respectively [19, 31], one of which compared diclofenac-orphenadrine to piroxicam and to placebo for postoperative pain in patients undergoing knee arthroscopy. This study by Málek et al. showed a significantly lower number of additional pain medication needed in the diclofenac-orphenadrine group when compared to placebo as well as lower average postoperative pain scores. Interestingly, also a lower number of side effects occurred in the diclofenac-orphenadrine group when compared to placebo. A third, Austrian study, conducted by Gombotz et al., was a multicenter double-blind randomized study comparing orphenadrine-diclofenac to placebo after total hip arthroplasty (THA) after spinal anesthesia using bupivacaine. Gombotz et al. were able to report a significant reduction on PCA analgesics used over 24 h postsurgery in the diclofenac-orphenadrine group when compared to placebo [21]. There are several key differences between the study by Gombotz et al. and this study, ultimately making a comparison difficult. First, it is difficult to compare THA with ligament surgery since there are relevant differences in patient demographics and postoperative pain profile. Preoperative pain, which is also a predictive factor of postoperative pain intensity was almost non-existent in our cohort with a mean level of 1.4 mm VAS (on a 100 mm scale!); preoperative pain assessment was not reported by Gombotz et al. but is presumably higher considering that the main indication for a THA is rest pain, pain with activity and functional limitations in patients with primary osteoarthritis [32, 33]. Mean patient age differed greatly between the 2 studies with 63 years compared to 32 years in our cohort; younger age is an independent predictor of greater postoperative pain [34]. Furthermore, Gombotz et al. assessed patients receiving spinal anesthesia in comparison to total intravenous anesthesia in our study. Differences in postoperative pain progression, specifically less pain in the first 2h with spinal anesthesia which then changes into greater pain after several hours postsurgery are described when comparing it to total intravenous anesthesia [35].

Orphenadrine binds to the phencyclidine (PCP) binding site of the NMDA receptor which has been shown to delay the emergence and maintenance of mechanical and thermal hyperalgesia and allodynia [16, 36–38]. Additionally, Schaffler et al. provided evidence for an analgesic effect via a central mechanism for orphenadrine alone as well as for the combination with diclofenac in a human pain model [16]. Still, the existence and the physiological mechanisms behind opioid-induced hyperalgesia (OIH) are not entirely clear. One commonly proposed mechanism is sensitization of spinal neurons leading to enhanced nociception, a mechanism mediated by the central glutaminergic system via the NMDA receptors, and which can be reversed by NMDA receptor antagonists. Positive effects of interoperative ketamine on remifentanil-induced hyperalgesia have been described and have been attributed to its antagonistic binding of the NMDA receptors [39, 40]. Therefore, we were anticipating an additional opioid-sparing effect when using diclofenac-orphenadrine compared to diclofenac only. Such a finding would make diclofenac-orphenadrine a valuable option in multimodal pain management especially when using remifentanil intraoperatively. With no difference in opioid requirements and pain intensity between the two groups we suspect that OIH has not occurred, although no specific OIH testing for allodynia was performed [41].

The most extensively discussed AEs in the literature on diclofenac are renal dysfunction, cardiovascular events, bleeding and thrombophlebitis, all of which occur infrequently and at similar rates as in other NSAIDs [4, 8]. Common side effects of orphenadrine are blurred vision, confusion, delirium or hallucinations, drowsiness, constipation, urination retention, dry eyes, mouth, nose, or throat and can be explained by its anticholinergic properties but occurred almost exclusively in higher doses as prescribed for Parkinson treatment and in older patients [24, 25, 42]. Even withdrawal symptoms have been described after extended prescriptions [43]. Concerning the central anticholinergic properties, none of the included patients tested positive for delirium in this study. In summary, our findings regarding safety parameters for diclofenac and diclofenac-orphenadrine are in line with the literature and further verify a tolerability of diclofenac and diclofenac-orphenadrine similar to that of placebo in a healthy population. However, our study was not powered to evaluate drug safety and tolerability and therefore would potentially miss rare adverse events.

An additional limitation of this study is the lack of pain assessment during joint movement. A study by Cho et al. on perioperative use of pregabalin failed to detect a reduction in postoperative opioid demand in patients undergoing cruciate ligament surgery but resulted in a reduction in pain during joint movement [44].

It seems possible that there is an opioid-sparing effect of orphenadrine-diclofenac which we failed to detect because of insufficient power. The effect size for the sample size analysis may have been overestimated, and more patients per group likely might have been needed. Finally, a significant difference for PCA analgesic consumption between placebo and diclofenac/orphenadrine-diclofenac was detected after removal of two datapoint outliers resulting from two patients excessively pressing their respective PCA pumps, while both reporting low pain levels post hoc. However, as we cannot exclude that this particular PCA activation was due to pain and not “PCA misuse” this result needs to be interpreted with caution and is exploratory.

An additional limitation of our study is the lack of evaluation of further variables that might influence pain perception such as social and cultural background as well as ethnicity [45, 46].

## Conclusion

In this study orphenadrine-diclofenac did not significantly reduce postoperative opioid requirements when compared to diclofenac or placebo. However, an exploratory analysis without two patients, who incessantly pressed their PCA buttons while reporting low pain levels, combining the orphenadrine-diclofenac and diclofenac only group showed a reduction of postoperative opioid requirements when compared to placebo. Additionally, orphenadrine-diclofenac has a similar side effect profile when compared to diclofenac alone. Due to its pharmacological properties orphenadrine-diclofenac could have potential benefits concerning OIH as well as postoperative mobilization. Further research is required to determine a possible benefit of adding orphenadrine to diclofenac in postoperative and nonsurgical pain management as part of a multimodal approach as well as a possible prophylaxis against OIH.
